# Supplementary material for: Extracellular calcium functions as a molecular glue for transmembrane helices to activate the scramblase Xkr4
Source: Nat Commun. 2023 Sep 11;14:5592. doi: 10.1038/s41467-023-40934-2 (PMC10495444; doi:10.1038/s41467-023-40934-2)
Supplement: Supplementary file 1 — Supplementary Information [file 41467_2023_40934_MOESM1_ESM.pdf]

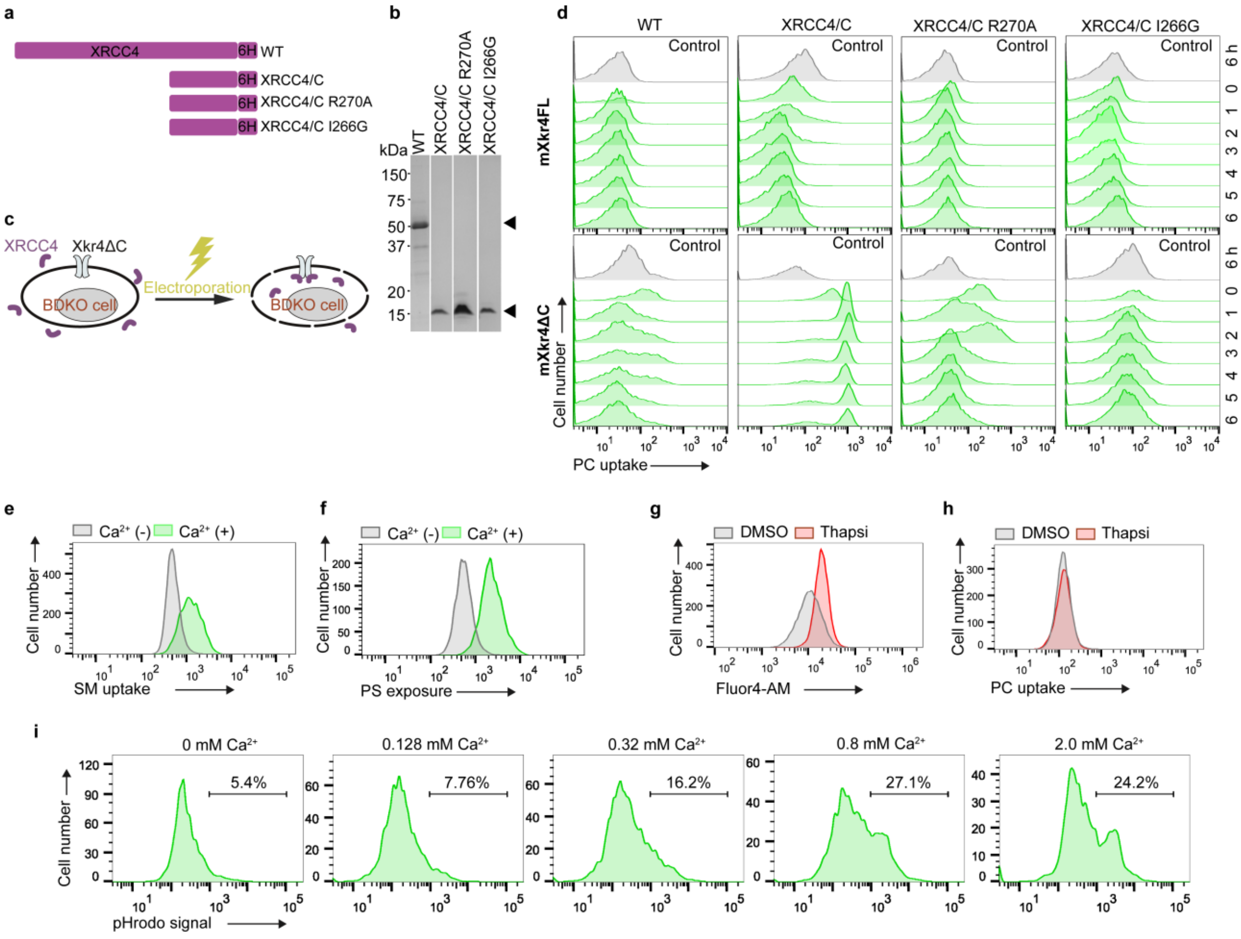

Supplementary Figure 1: Activation of Xkr4 by XRCC4 mutants. **a** Schematic representation of purified XRCC4 and its mutants. **b** SDS-PAGE of purified XRCC4 and its mutants. The proteins were purified from *E. coli* using Glutathione sepharose and incubated with recombinant enterokinase. The cleaved XRCC4 (XRCC4/C) was purified using a nickel column and applied to size-exclusion chromatography. Purified XRCC4/C proteins were applied to SDS-PAGE and stained with CBB. XRCC4/C data was used for Fig. 1b. **c** Schematic representation of XRCC4 FL (WT) and XRCC4/C introduction by electroporation. **d** Effect of purified XRCC4 and its mutants on PC uptake. XRCC4 and mutants were introduced into BDKO cells expressing Xkr4FL or Xkr4ΔC by electroporation. Control: electroporation without XRCC4. **e** Sphingomyelin (SM) uptake assay of BDKO cells expressing aXkr4. Cells were incubated with NBD-SM in the presence or absence of 1 mM Ca^{2+} for 40 min. **f** PS exposure assay of BDKO cells expressing aXkr4. Cells were incubated with EGFP-fused MFG-E8 in the presence or absence of 1 mM Ca^{2+} for 40 min. **g** Thapsigargin (Thapsi) treatment on calcium increase. BDKO cells expressing aXkr4 were treated with thapsi at 1 μM or the same volume of DMSO in the presence of 0.5 mM EGTA. Inner Ca^{2+} concentration was analyzed by FACS using 4 μM Fluo4-AM. **h** Thapsi treatment on PLS. BDKO cells expressing aXkr4 were treated with 1 μM thapsi or the same volume of DMSO. Cells were then applied to the PC uptake assay. **i** Engulfment of apoptotic cells by macrophages. Xkr4-expressing PLB cells were stimulated with UV at 2000 J/m^2 and labeled with pHrodo. Labeled apoptotic cells were incubated with thioglycolate-elicited macrophages in the presence of an indicated concentration of Ca^{2+} for 4 h and analyzed by FACS. Source data are provided as Source Data file.

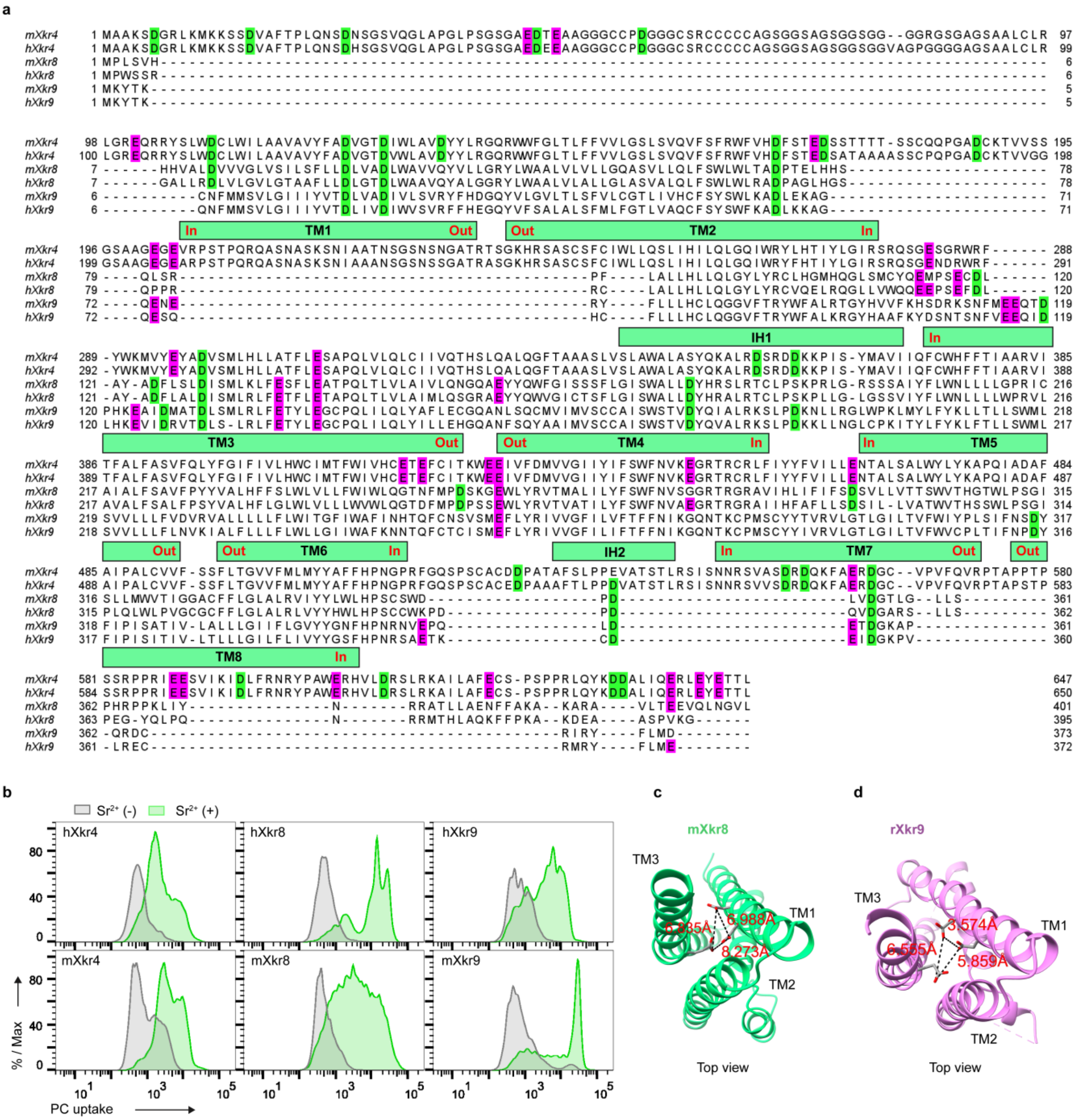

Supplementary Figure 2: Activation of Xkr family membranes by Sr^{2+}. a Sequence alignment of human and mouse Xkr4, Xkr8, and Xkr9. Highly conserved acidic residues among Xkr4, Xkr8, and Xkr9 are highlighted in green for Asp and purple for Glu. The potential calcium-binding residues were labeled with a red character numbered in mXkr4 position. **b** PC uptake assay of human and mouse Xkr4, Xkr8, and Xkr9. BDKO cells expressing human or mouse Xkr family of these three were treated with a final concentration of 1.0 mM $SrCl_2$ after apoptotic stimulation with UV. PLB cells expressing Xkr4 were treated with 2000 J/m^2, and Xkr8 and Xkr9 were treated with 200 J/m^2. **c** A top view of mouse Xkr8 (PDB: 7DCE). The potential calcium-binding residues were highlighted as sticks (oxygen: red), and the distances of each residue were represented by dash lines. **d** A top view of rat Xkr9 (PDB:7P16). The potential calcium-binding residues as sticks (oxygen: red), and the distances of each residue were represented by dash lines.

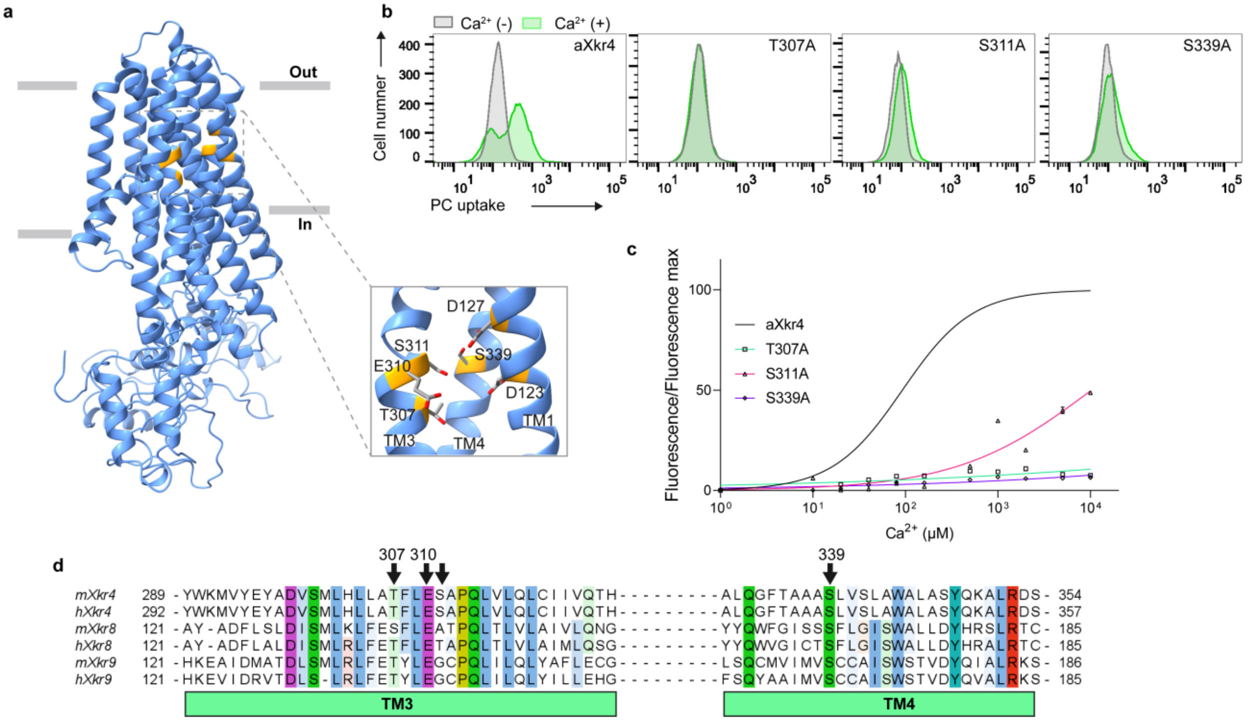

Supplementary Figure 3: Non charged residues present in the calcium-binding site. a A Ca^{2+}-binding site of mXkr4. The calcium-binding site-forming amino acids were shown as sticks (oxygen: red). **b** PC uptake assay of BDKO cells expressing aXkr4 with single mutations. BDKO cells expressing each mutant (T307A, S311A, or S339A) were incubated with NBD-PC at a final concentration of 0.125 mM Ca^{2+} or without it, and applied to FACS. **c** Dose-response curves of Xkr4 activity based on NBD-PC uptake. BDKO cells expressing aXkr4 or Ala mutant (T307A, S311A, and S339A), were incubated with NBD-PC at various Ca^{2+} concentrations and analyzed by FACS. Averages of triplicates were shown with SD. The result of aXkr4 in **c** are the same as Fig. 4b. **d** The sequence alignment of human and mouse Xkr4, Xkr8, and Xkr9. The conserved sequence was colored. Calcium-binding sites in TM3 and TM4 were indicated by arrows with amino acid positions according to mXkr4. Source data are provided as Source Data file.

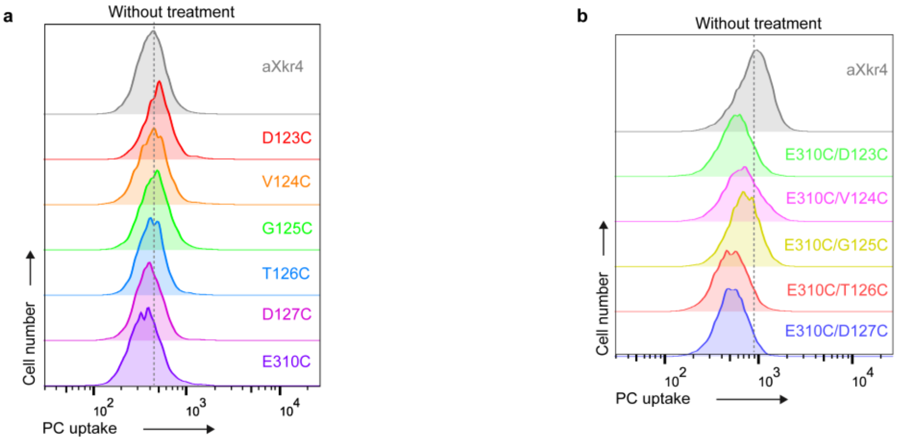

Supplementary Figure 4: Cys mutants of aXkr4. a PC uptake assay of Cys single mutations. BDKO cells expressing aXkr4 containing Cys single mutations without the oxidative reagent treatment. Cells were incubated with NBD-PC without Ca^{2+}. **b** PC uptake assay of Cys double mutations. BDKO cells expressing aXkr4 containing Cys double mutations without the oxidative reagent treatment. Cells were incubated with NBD-PC without Ca^{2+}.

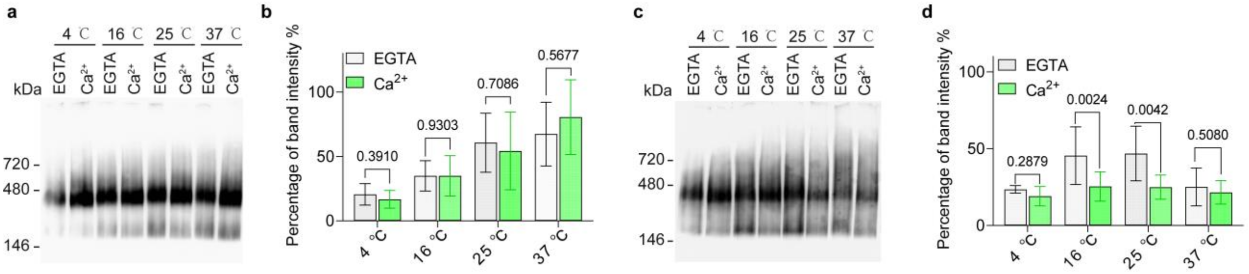

Supplementary Figure 5: Xkr4 stability. a-d Thermostability of Xkr4. Cells expressing V5 and EGFP-fused Xkr4ΔC (**a, b**) or aXkr4 (**c, d**) were solubilized with 0.5% GDN and 0.5% LMNG in the presence of 1 mM Ca^{2+} or 0.5 mM EGTA. The dimer fraction of Xkr4 was collected by size exclusion chromatography, incubated at 4, 16, 25, and 37 °C for 1 h, and then applied to BN-PAGE, followed by detection of Xkr4 with anti-V5-HRP antibody (**a, c**). The percentage of monomers was calculated and shown as averages of triplicates on the right with SD (**b, d**). n = 3 samples for each condition. Statistical test was performed using a two-tailed Student's t test. $p<0.05$ was considered statistically significant. Source data are provided as Source Data file.

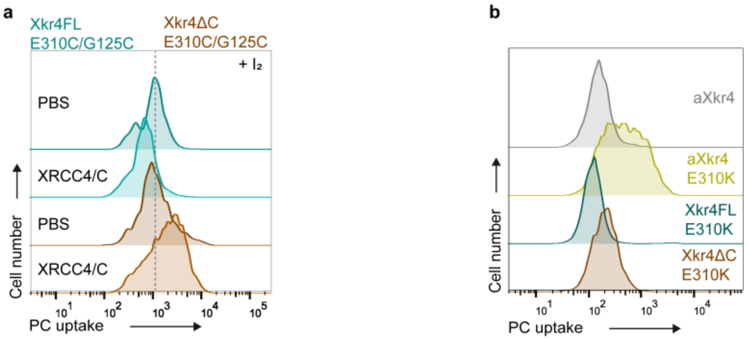

Supplementary Figure 6: Xkr4 activation by induced disulfide bond or salt bridge. a Xkr4 activation in Cys mutation. Cells expressing Xkr4FL G125C/E310C or Xkr4ΔC G125C/E310C were electroporated in the presence of 0.5 µM XRCC4/C or PBS buffer for control and were treated with a final concentration of 25 µM I_2 in TBS buffer at room temperature for 10 min. PC uptake assay was analyzed by FACS. **b** Xkr4 activation in Lys mutation. BDKO cells expressing aXkr4, aXkr4 E310K, Xkr4FL E310K, or Xkr4ΔC E310K were applied to PC uptake assay without Ca^{2+}.

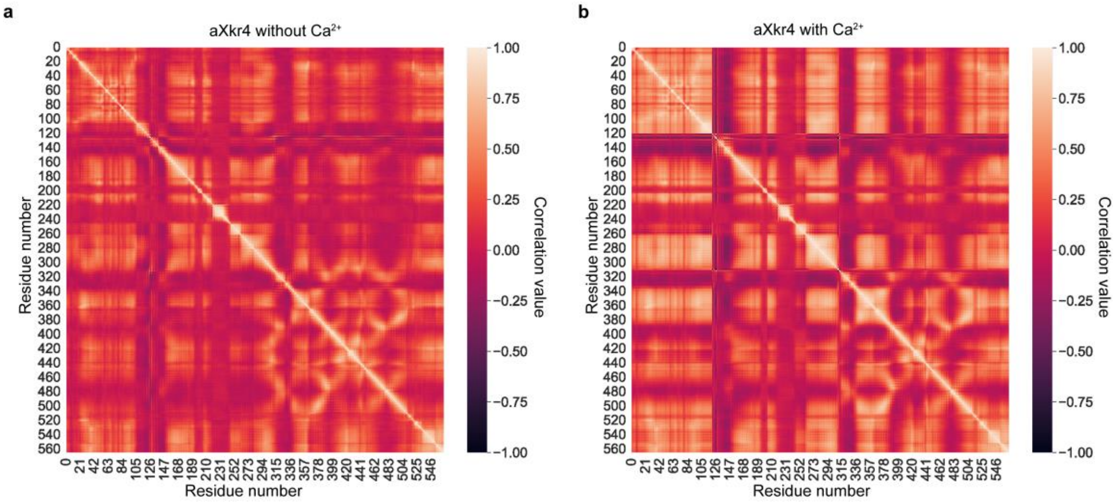

Supplementary Figure 7: Molecular dynamics simulation of Xkr4 with Ca^{2+}. a, b Correlation matrix of distance changes of each pair of amino acids. After performing MD simulation and analyzing the trajectories of all amino acids, we calculated how each pair of amino acids changes the distances between the amino acids and the Ca^{2+} position which is postulated to locate at the center of D123, D127, and E310. **a** Correlation matrix of aXkr4 without Ca^{2+}. **b** Correlation matrix of aXkr4 with Ca^{2+} bridge. To mimic Ca^{2+} binding, we applied " Ca^{2+} bridge".

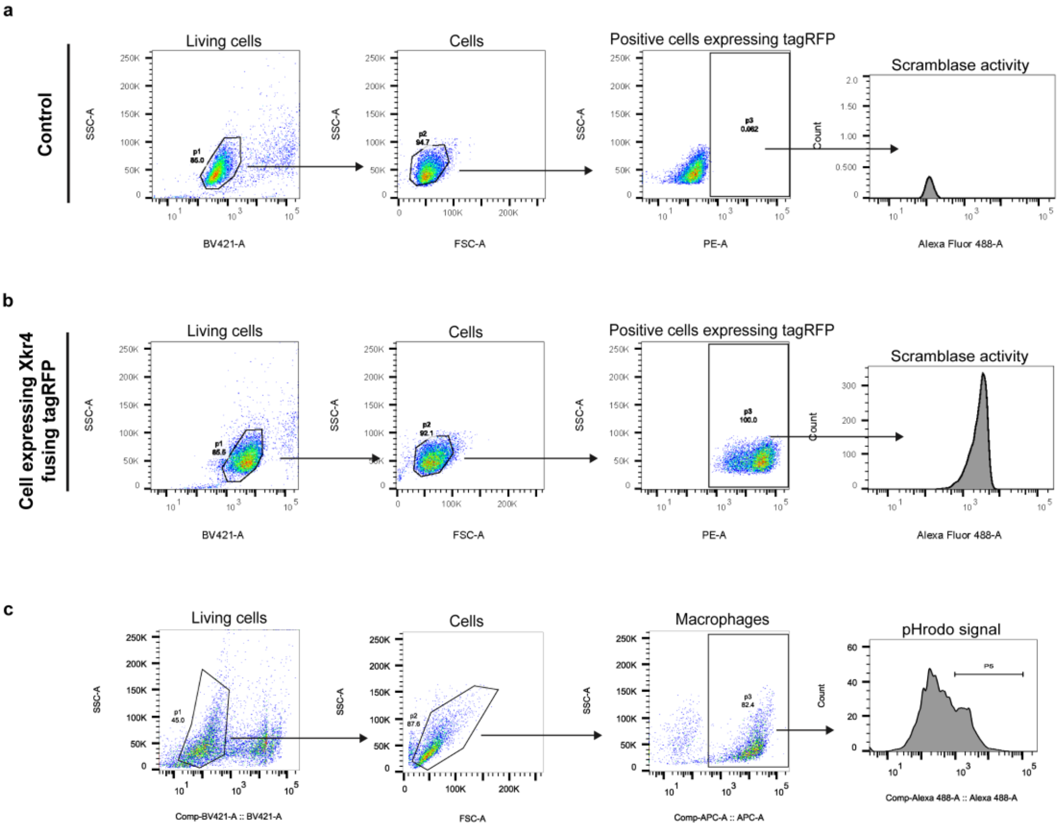

Supplementary Figure 8: Gating strategies. a, b Gating strategy for scramblase activity assay. In BDKO cells, dead cells were excluded, labeled with DAPI, and cell debris were excluded using SSC-A and FSC-A. The population of tagRFP-fused Xkr4 was used for analysis of phospholipid scrambling activity by NBD-PC uptake, NBD-SM uptake, or PS exposure. **a** and **b** represent cells without or with tagRFP-fused Xkr4, respectively. **c** Gating strategy for engulfment of apoptotic cells by macrophages. Macrophages were labeled with DAPI to exclude the dead cells and exclude the debris based on SSC-A and FSC-A. CD11b-positive cells were identified as macrophages and subsequently analyzed using pHrodo signal for engulfment of apoptotic cells.
