# Supplementary material for: Extracellular calcium functions as a molecular glue for transmembrane helices to activate the scramblase Xkr4
Source: Nat Commun. 2023 Sep 11;14:5592. doi: 10.1038/s41467-023-40934-2 (PMC10495444; doi:10.1038/s41467-023-40934-2)
Supplement: Supplementary file 3 — Description of Additional Supplementary files [file 41467_2023_40934_MOESM3_ESM.docx]

Legend for Supplementary Software

Molecular Dynamics code used in the manuscript provided as .dcd files. File 1 corresponds to MD analysis with calcium bridge and file 2 to MD analysis without calcium bridge. The correlation matrix analysis is provided as a .txt file.
