# Supplementary material for: Extracellular calcium functions as a molecular glue for transmembrane helices to activate the scramblase Xkr4
Source: Nat Commun. 2023 Sep 11;14:5592. doi: 10.1038/s41467-023-40934-2 (PMC10495444; doi:10.1038/s41467-023-40934-2)
Supplement: Supplementary file 4 — Reporting Summary [file 41467_2023_40934_MOESM4_ESM.pdf]

nature portfolio

Corresponding author(s): Jun Suzuki

Last updated by author(s): Aug 2, 2023

Reporting Summary

Nature Portfolio wishes to improve the reproducibility of the work that we publish. This form provides structure for consistency and transparency in reporting. For further information on Nature Portfolio policies, see our Editorial Policies and the Editorial Policy Checklist.

Statistics

For all statistical analyses, confirm that the following items are present in the figure legend, table legend, main text, or Methods section.

n/a	Confirmed	
☐	☒	The exact sample size (*n*) for each experimental group/condition, given as a discrete number and unit of measurement
☐	☒	A statement on whether measurements were taken from distinct samples or whether the same sample was measured repeatedly
☐	☒	The statistical test(s) used AND whether they are one- or two-sided<br>*Only common tests should be described solely by name; describe more complex techniques in the Methods section.*
☒	☐	A description of all covariates tested
☐	☒	A description of any assumptions or corrections, such as tests of normality and adjustment for multiple comparisons
☐	☒	A full description of the statistical parameters including central tendency (e.g. means) or other basic estimates (e.g. regression coefficient) AND variation (e.g. standard deviation) or associated estimates of uncertainty (e.g. confidence intervals)
☐	☒	For null hypothesis testing, the test statistic (e.g. *F*, *t*, *r*) with confidence intervals, effect sizes, degrees of freedom and *P* value noted<br>*Give P values as exact values whenever suitable.*
☒	☐	For Bayesian analysis, information on the choice of priors and Markov chain Monte Carlo settings
☒	☐	For hierarchical and complex designs, identification of the appropriate level for tests and full reporting of outcomes
☒	☐	Estimates of effect sizes (e.g. Cohen's *d*, Pearson's *r*), indicating how they were calculated

Our web collection on statistics for biologists contains articles on many of the points above.

Software and code

Policy information about availability of computer code

Data collection	FACS Diva and BD FACSuite v1.21 for flow cytometry analysis. CafeMol 3.2.1 for molecular dynamic analysis.
Data analysis	GraphPad PRISM 9.2.0 for affinity of calcium analysis, Illustrator V 26.0.3 for figure drawing, FlowJo for flow cytometry analysis, Chimera 1.16 and Chimera X 1.4 for structure analysis, Jalview 2.11.1.7 for sequence alignment, Modeller 10.1 for structure prediction, Matplotlib 3.5.1 and Numpy for data analysis of membrane tension.

For manuscripts utilizing custom algorithms or software that are central to the research but not yet described in published literature, software must be made available to editors and reviewers. We strongly encourage code deposition in a community repository (e.g. GitHub). See the Nature Portfolio guidelines for submitting code & software for further information.

Data

Policy information about availability of data

All manuscripts must include a data availability statement. This statement should provide the following information, where applicable:

- Accession codes, unique identifiers, or web links for publicly available datasets
- A description of any restrictions on data availability
- For clinical datasets or third party data, please ensure that the statement adheres to our policy

Previously published cryo-EM models of mXkr8 and rXkr9, used in this study, are available in the Protein Data Bank (PDB) under the PDB ID 7DCE and 7P16. All the

data produced or analyzed in this study has been incorporated into this article and its supplementary files. Source data are provided in this paper. The MD simulations codes have been provided as supplementary files.

Human research participants

Policy information about studies involving human research participants and Sex and Gender in Research.

Reporting on sex and gender	n/a
Population characteristics	n/a
Recruitment	n/a
Ethics oversight	n/a

Note that full information on the approval of the study protocol must also be provided in the manuscript.

Field-specific reporting

Please select the one below that is the best fit for your research. If you are not sure, read the appropriate sections before making your selection.

☒ Life sciences ☐ Behavioural & social sciences ☐ Ecological, evolutionary & environmental sciences

For a reference copy of the document with all sections, see nature.com/documents/nr-reporting-summary-flat.pdf

Life sciences study design

All studies must disclose on these points even when the disclosure is negative.

Sample size	In the case of membrane tension experiments, we observed the cells under 100× oil objective, confirmed the cell morphology, and randomly selected 10 cells because variations among single cells can be determined using around 10 cells in general. In the case of flow cytometry, 10,000 cells were analyzed because this number is sufficient to clearly show data in histograms.
Data exclusions	We exclude a few cell analysis of membrane tension experiments in which cells showed abnormal structure.
Replication	The experiments for statistic analysis were repeatedly examined for more than three time for FACS analysis.
Randomization	In most of experiments, we used Ba/F3 cells deficient in both Xkr8 and TMEM16F because we can solely analyze functions of Xkr4 on plasm membrane. In FACS analysis, we quantified the fluorescence of 10,000 cells, indicating no bias to select specific cells.
Blinding	In FACS analysis, we analyzed each sample in the same voltage and amplitude, and we did not change the parameters according to samples. in addition, 10,000 cells were analyzed without bias. Therefore, blinding is not necessary for our FACS analysis.

Reporting for specific materials, systems and methods

We require information from authors about some types of materials, experimental systems and methods used in many studies. Here, indicate whether each material, system or method listed is relevant to your study. If you are not sure if a list item applies to your research, read the appropriate section before selecting a response.

Materials & experimental systems

n/a	Involved in the study	
☐	☒	Antibodies
☐	☒	Eukaryotic cell lines
☒	☐	Palaeontology and archaeology
☐	☒	Animals and other organisms
☒	☐	Clinical data
☒	☐	Dual use research of concern

Methods

n/a	Involved in the study	
☒	☐	ChIP-seq
☐	☒	Flow cytometry
☒	☐	MRI-based neuroimaging

Antibodies

Antibodies used	Anti-V5-HRP using 6000 dilution, Thermo Fisher Scientific, Cat# R961-25. Anti-mouse basigin using a final concentration of 0.1 μg/ml,

Antibodies used	Biolegend, Cat# 123701. Anti-rat IgG biotin using a final concentration of 0.2 µg/ml, Thermo Fisher Scientific, Cat# 31830. APC anti-mouse/human CD11b using 400 dilution, Biolegend, cat#101211.
Validation	Anti-V5-HRP, mouse monoclonal antibody to detect the Xkr4 tagged with N-terminal V5 in BN-PAGE analysis.<br>Anti-mouse basigin, mouse monoclonal antibody to detect the CD147 expressing in Ba/F3 cells for membrane tension experiments.<br>Anti-mouse/human CD11b, rat monoclonal antibody to detect the CD11b expressing in macrophage for engulfment experiments.

Eukaryotic cell lines

Policy information about cell lines and Sex and Gender in Research

Cell line source(s)	PLB cells (woman) were gifted from Prof. W. Hiraoka.<br>Ba/F3 cells deficient in both Xkr8 and TMEM16F were previously generated (Maruoka et al., 2021 Mol Cell).<br>HEK293T cells, n/a<br>CHO cells, n/a.
Authentication	PLB cells can be authenticated by no Xkr8 expression and differentiation by DMSO treatment.<br>Ba/F3 cells can authenticated by IL3-dependency.<br>HEK293T cells can authenticated by morphology and high transfection efficiency.<br>CHO cells cab authenticated by morphology.
Mycoplasma contamination	At the beginning, cells were cultured with MC-210 (WAKEN) to exclude potential micoplasma contamination and tested negative for micoplasma contamination.
Commonly misidentified lines (See ICLAC register)	No commonly misidentified lines were used in this study.

Animals and other research organisms

Policy information about studies involving animals; ARRIVE guidelines recommended for reporting animal research, and Sex and Gender in Research

Laboratory animals	C57BL6/J, 8-weeks-old female mouse was used in this study.
Wild animals	n/a
Reporting on sex	Female mouse was used for collection of thioglycollate-elicited peritoneal macrophages.
Field-collected samples	n/a
Ethics oversight	Animal study protocols were approved by Committee on the Animal Experiments of Kyoto University (Approved Number:49-4).

Note that full information on the approval of the study protocol must also be provided in the manuscript.

Flow Cytometry

Plots

Confirm that:

- [x] The axis labels state the marker and fluorochrome used (e.g. CD4-FITC).
- [x] The axis scales are clearly visible. Include numbers along axes only for bottom left plot of group (a 'group' is an analysis of identical markers).
- [x] All plots are contour plots with outliers or pseudocolor plots.
- [x] A numerical value for number of cells or percentage (with statistics) is provided.

Methodology

Sample preparation	Cell lines were used as samples to analyze lipid scrambling activity. Macrophages were prepared from peritoneum after injection of 2 ml of 3% thioglycolate.
Instrument	BD FACS ARIA2 and BD FACSLyric
Software	FACS Diva for BD FACS ARIA2<br>BD FACSuite v1.21 for BD FACSLyric
Cell population abundance	Cell lines were used, and therefore we consider cell population abundance is 100%.
Gating strategy	SSC-A/BV421-A (DAPI) to gate the living cells.<br>SSC-A/FSC-A to gate the cell size and complexity.

SSC-A/PE-A to separate the cells expressing target proteins fused with tagRFP.
Histogram/ Alexa Fluor 488-A to quantify the fluorescence of NBD-PC.

☒ Tick this box to confirm that a figure exemplifying the gating strategy is provided in the Supplementary Information.
